# Arsenic as a Dental Therapeutic Agent

**Published:** 1845-12

**Authors:** 


*Arsenic as a Dental Therapeutic Agent.*—We would direct the attention of our readers to an article in another part of the present number of the Journal, on the use of arsenic as a remedy for tooth-ache, and for the destruction of an exposed dental pulp, with a view to the subsequent preservation of the tooth by filling, and ask for it a careful and attentive perusal. During the last five or six years, arsenic has been more generally and extensively employed for the above named purposes than any other remedy or therapeutic agent, and at first it promised to be of great value; but before we had ever used it, we expressed our disapprobation to its employment for these purposes, for the reason, that a tooth after having been deprived of its vitality, could not, in accordance with a well known law of the animal economy, remain in the mouth with impunity; that if it did not immediately give rise to the formation of an alveolar abscess, it would of necessity exercise a morbid influence upon the circumjacent living parts. Soon after we had expressed this opinion, however, we were assured by some of the first practitioners in New York and Philadelphia, that it was fully realizing the expectations of all who advocated its use; and, willing, for the time, to yield an opinion based upon mere pathological reasoning, when opposed to so formidable an array of alleged facts, we determined to give it a fair trial. At first it promised to be an invaluable acquisition to the resources of the dental branch of medicine. It acted almost like a charm—cured tooth-ache, when resulting from the exposure of the pulp of the tooth, by destroying the vitality of this partof the organ, and we were forced, for a time, to believe that our former reasoning upon the subject was based upon a false hypothesis.

But the deception did not last long: the effects which we had anticipated previously to using it, soon began to manifest themselves—first in chronic inflammation of the alveolo dental periosteum, afterwards in pain and soreness of the tooth, and ultimately, in a large majority of cases, in alveolar abscess. It was contended by some practitioners, that these subsequent morbid developments were the result of irritation produced by an accumulation of matter in the root or roots, if the tooth had more than one of the organ, and that by filling these, they might be prevented. But neither by this nor any other plan of treatment, have we, on an average, been able to prevent them in more than one case out of five, and we have no doubt, that sooner or later, in the apparently successful cases, the effects above described will manifest themselves. The reason that they have not done so in these as soon as they did in the others, is doubtless owing to the fact, that the alveolo dental membrane in the former cases, was less susceptible to the action of morbid irritants, than it was in the latter, or that the immediate effect produced on it by the arsenic was less powerful. We have had occasion to extract many teeth which had been treated in this manner, not only by ourself, but by other practitioners.

This is the result of our own experience with regard to the use of arsenic for destroying the nerves of teeth, preparatory to the operation of filling, and so far as we have been able to obtain the views of others upon the subject, we are happy to believe, that this most objectionable of all species of dental practice, is almost wholly abandoned by respectable practitioners throughout the length and breadth of the land.

As to the other effects spoken of by our associate Dr. Westcott, as liable to result from the employment of this agent, we do not think there is any danger of their occurring, if it be applied by a skilful practitioner ; but unfortunately, its use is now chiefly confined to those who have the most meagre claims to skill in the profession. In the hands of such, its use is certainly attended with much danger. A case was mentioned to us a few days since, of a little girl, about twelve years of age, who was taken to a self-styled dentist of this city for the purpose of obtaining relief from the tooth-ache. The cavity, we believe, was in the side of the tooth, and the remedy was applied on a little raw cotton, which not having been properly secured, got between the teeth in such a manner as to come in contact with the side of the tongue, producing, not only a most hideous ulcer, but it also came very near destroying the life of the child. Skilful medical aid was immediately called in, but it was three months before she completely recovered. This is only one of many cases, which we might mention of the serious effects which have resulted from the employment of arsenic as a remedy for tooth-ache. The use of this agent, therefore, being attended, not only with danger, but also failing to secure the preservation of the teeth to which it is applied, its employment for this purpose, should at once and forever, be abandoned. But we fear, that so long as advertising to cure the tooth-ache in two or five minutes, can be made profitable, it will continue to be used.—*Balt. Ed.*

